# Supplementary material for: Level of Acceptance of Mandatory Vaccination and Legal Sanctions for Refusing Mandatory Vaccination of Children
Source: Vaccines (Basel). 2022 May 20;10(5):811. doi: 10.3390/vaccines10050811 (PMC9146574; doi:10.3390/vaccines10050811)
Supplement: Supplementary file 1 [file vaccines-10-00811-s001.zip › vaccines-1682711-supplementary.pdf]

P1. There are currently a number of different opinions on vaccination. Do you agree or disagree with the following statements?
[SCRIPT: item question, KAFETERIA: 4. strongly agree, 3. rather agree, 2. rather disagree, 1. strongly disagree, 98. hard to say, 99. refuse to answer (Interviewer: do not read)]

1. In general, vaccinating children causes more good than bad.
2. In the first years of life, children receive too many vaccines.
3. Vaccines for children are safe
4. Vaccines for children can cause serious side effects, complications
5. Vaccination is the most effective way to protect children from serious diseases
6. Thanks to childhood immunisation, many dangerous diseases are now virtually non-existent
7. Parents of vaccinated children are sufficiently informed about the side effects of vaccines
8. Vaccines for children can cause serious developmental disorders such as autism
9. Vaccinations are promoted not because they are actually needed, but because it is in the interests of the pharmaceutical companies
10. Instead of vaccination, it is better to let the child suffer from an infectious disease, because it is nothing terrible - a few days of being ill with measles, smallpox or rubella and we already have natural immunity for life
11. Vaccination is compatible with my religious beliefs
12. Medical experts appearing in the media encouraging vaccination are credible to me
13. Vaccinations weaken a child's natural immunity

P2. Year on year, there is a growing number of parents who do not vaccinate their children against infectious diseases for fear of vaccines. In the discussion on this topic, arguments are made about parents' rights to decide, on the one hand, and about the risk of the return of many serious infectious diseases, on the other. Which of the following positions is closer to you?

14. No preventive vaccinations should be compulsory and the decision on whether or not to vaccinate a child should be entirely up to the parents.
15. Immunization against the most dangerous infectious diseases should be mandatory, and parents can decide only on possible additional vaccinations.
16. I have a different opinion
    99. refusal to reply (Interviewer: do not read)

P3. In general, most people in your immediate environment believe that:

17. Immunization against the most dangerous infectious diseases should be mandatory, and parents can decide only on possible additional vaccinations.
18. Preventive vaccinations should be purely voluntary and the need for each vaccination should be decided by parents
19. Vaccination against infectious diseases is harmful and should be banned.
20. I don't know, this topic is not discussed

P4. Do you think there should be legal or financial consequences for people who avoid vaccinating their children?

4. definitely yes
3. rather yes

2 Rather not [SKRYPT: filter: go to question 13].
1. definitely not [SKRYPT: filter: go to question 13].

97 I have no opinion on this [SKRYPT: filter: go to question 13].
99 Refused to answer (Interviewer: do not read)[SKRYPT: filter: go to question 13].

P5. [SKRYPT: post-filter question] Which of the following consequences for vaccine avoiders do you consider acceptable? [SKRYPT: multiple choice]

1. Refusal of non-vaccinated children to participate in extra-curricular activities organised by public institutions

2. Refusal of unvaccinated children to participate in organised holidays
3. Refusal to admit an unvaccinated child to a public nursery or kindergarten
4. Fines for parents/guardians
5. Forced vaccination of a child
6. Imprisonment of parent/guardian
7. None of the above

99. refusal to reply (Interviewer: do not read)

P6. When talking to your doctor about vaccination, what type of information would most convince you to get vaccinated?

1. Presentation of science-based arguments
2. Provide arguments based on the medical experience of your doctor
3. Providing information on whether the doctor has himself vaccinated and/or whether his family has been vaccinated
4. No argument would convince me to get vaccinated

99. refusal to reply (Interviewer: do not read)
